# The Synergetic Impact of Anionic, Cationic, and Neutral Polymers on VES Rheology at High-Temperature Environment

**DOI:** 10.3390/polym14061145

**Published:** 2022-03-13

**Authors:** Amro Othman, Mohammed AlSulaimani, Murtada Saleh Aljawad, Shiv Shankar Sangaru, Muhammad Shahzad Kamal, Mohamed Mahmoud

**Affiliations:** 1 Department of Petroleum Engineering, King Fahd University of Petroleum & Minerals, Dhahran 31261, Saudi Arabia; g201904750@kfupm.edu.sa (A.O.); mmahmoud@kfupm.edu.sa (M.M.); 2 Baker Hughes, Dhahran 31952, Saudi Arabia; m.alsulaimani@outlook.com; 3 Center for Integrative Petroleum Research, King Fahd University of Petroleum & Minerals, Dhahran 31261, Saudi Arabia; shahzadmalik@kfupm.edu.sa

**Keywords:** polymer, rheology, VES, synergy

## Abstract

Hydraulic fracturing operations target enhancing the productivity of tight formations through viscous fluid injection to break down the formation and transport proppant. Crosslinked polymers are usually used for desired viscoelasticity of the fracturing fluid; however, viscoelastic surfactants (VES) became a possible replacement due to their less damaging impact. To design a fracturing fluid with exceptional rheological and thermal stability, we investigated mixing zwitterionic VES with carboxymethyl cellulose (CMC), hydroxyethylcellulose (HEC), or a poly diallyl dimethylammonium chloride (DADMAC) polymers. As a base fluid, calcium chloride (CaCl_2_) solution was prepared with either distilled water or seawater before adding a polymer and the VES. A Chandler high-pressure, high-temperature (HPHT) viscometer was used to conduct the viscosity measurements at a shear rate of 100 1/s. It has been found that adding 1% CMC polymer to 9% (*v*/*v*) VES increases the viscosity more compared to 10% (*v*/*v*) VES at reservoir temperatures of 143.3 °C. On the other hand, adding only 1.0% of HEC to 9% (*v*/*v*) VES doubled the viscosity and proved more effective than adding CMC. HEC, nevertheless, reduced the system stability at high temperatures (i.e., 148.9 °C). Adding DADMAC polymer (DP) to VES increased the system viscosity and maintained high stability at high temperatures despite being exposed to saltwater. CaCl_2_ concentration was also shown to affect rheology at different temperatures. The improved viscosity through the newly designed polymer can reduce chemical costs (i.e., reducing VES load), making it more efficient in hydraulic fracturing operations.

## 1. Introduction

Hydraulic fracturing operations are applied to enhance the recovery of hydrocarbons from low permeability formations. Besides the increase in productivity, hydraulic fracturing can be used to control sand production from unconsolidated formations. Many factors impact fracture design, such as formation type, reservoir conditions, and the availability of freshwater, which depends on the location and cost. Hydraulic fracturing is designed through the selection of fluid additives, proppant size and type, injection rate, and treatment volume. During operations, the pad (i.e., clean fluid) is pumped to initiate the fracture then the slurry, consisting of proppant, is injected to propagate it further and keep it open under closure stresses. Hydraulic fracturing can improve production by establishing a conductive fracture to bypass local wellbore damage, extending the fracture to a considerable depth, and changing the reservoir fluid flow pattern [1]. The fluid viscosity must be optimized for hydraulic fracturing, considering the compatibility with the formation. Fracking fluids should be able to transport and suspend proppant and as well as create long fractures. Additives should be added to reduce the frictional losses and be ready to break and flow back at the end of the treatment [2,3].

The history of hydraulic fracturing started in 1947 as the first job was successfully completed in Kansas. Since then, millions of hydraulic fracturing operations have been performed worldwide, with the majority in the United States [4,5,6].

Water-based fracturing fluid has many limitations, including high cost, freshwater consumption, formation damage, and disposal issues [7,8,9]. Fracturing fluids are usually water-based; they include a gelling agent and a crosslinker to improve the viscosity. Slickwater is also a water-based fracturing fluid that consumes a large amount of freshwater [10]. Lately, the oil and gas industry has shown a strong interest in using seawater in hydraulic fracturing operations rather than freshwater. This is due to the high cost and scarcity of freshwater, besides the difficulties of freshwater transporting to offshore operations, which can be solved using seawater [11]. Using seawater, on the other hand, damages the formation due to the foreign ions existing in seawater and lowers the viscosity of polymer-based fluids. Numerous additives are employed to stabilize the fluid viscosity and reduce the formation damage. The viscoelastic surfactants (VES) exhibit better gelling behavior in the presence of such ions and hence could be utilized to increase the viscosity of seawater as an alternative to polymers and crosslinkers [10]. VES have shear-thinning viscoelastic behavior and leave no residue during flowback, utilizing internal and external breakers.

According to the charge in the hydrophilic portion, surfactants can be classified into anionic, nonionic, cationic, and zwitterionic surfactants. Anionic surfactants include perfluorooctane sulfonate (PFOS) and Dioctyl sodium sulfosuccinate (DOSS), nonionic surfactants include polyoxy ethylene glycol octyl phenol ethers (Triton X-100) and fatty methyl ester sulfonate (FMES), while cationic surfactants include cetyltrimethylammonium bromide (CTAB) and cetyltrimethylammonium chloride (CTAC). The Zwitterionic (amphoteric) surfactants contain both cationic and anionic portions as in sultaines which is 3-[(3-Cholamidopropyl) dimethyl ammonio]-1-propane sulfonate (CHAPS) [12,13]. Anionic surfactants have a high water-wetting ability in the sandstone reservoir, are less expensive, and can easily biodegrade compared to cationic surfactants. However, anionic surfactants suffer from temperature instability. Zwitterionic surfactants have higher temperature stability than other surfactants but are also more expensive. By combining two kinds of surfactants, the performance of surfactants may be enhanced synergistically [6,14].

The surfactants accumulate between oil and water, forming emulsions that are a function of micelles concentration. When the micelles concentration is not enough to form the barrier, the stable surfactants are called micro-emulsions. The micelles can be observed using transmission electrical microscopy (TEM), scanning electron microscope (SEM) to see the molecular orientation, and atomic force microscope (AFM) to obtain the viscoelastic properties [15]. VES fracturing fluids are characterized as micelles or vesicles based on the shape of surfactant aggregation in water. Both give an adequate viscosity for the system if the fracturing fluid is based solely on surfactant. The VES has a very low molecular weight in comparison to crosslinked polymers. It generates viscosity and forms worm-like micelles or vesicles in response to physical interactions and then loses this viscosity when the physical interaction changes. The aggregation size in vesicle-based VES is much larger than worm-like micelles, which results in a large surface area. It does not continuously lose and reform shape, making it more stable [16]. The micelles disintegrate into smaller spherical micelles and lose viscosity when it contacts organic and hydrophobic fluids such as oil and natural gas; as a consequence, there is no need for an extra breaker necessary for this system [13]. 

Water-soluble surfactants, in small concentrations, can form monomers when mixed with water and aggregate to form micelles at the critical concentration. Micelles are dynamic systems whose behavior depends on temperature and pressure. They have different shapes and can be characterized by geometry, salts, and temperatures. The viscoelastic behavior of the formed surfactants and the shape of micelles can be predicted from critical packing parameters calculated from the head group area, the volume of alkyl chains, and critical chain length. Micelles shapes play an essential role in determining the surface tension and the viscoelastic behavior of the host liquid. As the surfactant’s concentration increases, under controlled temperature, the surfactants form micelles at the critical concentration, then cylindrical groups, and finally Langmuir film. The shape changes occur within the water phase. This viscoelastic behavior can be controlled by adding compatible salts like sodium chloride (NaCl) and calcium chloride (CaCl_2_). Different effects were noticed due to different strengths of ion binding degree [6].

The stability of VES can be controlled by varying the temperature; increasing the temperature up to 40 °C can form an elongated micellar structure. The light also affects the VES properties with visible light resulting in vesicle structures with low viscosity and ultraviolet resulting in a worm-like micelle with higher viscosity. The VES viscosity can also be controlled by changing the concentration, adding polymers and nanoparticles, and through foam generation. All these factors can be either tracked by viscometer or rheology behavior [17]. 

At high surfactant concentrations, the micelles become warm-like, forming a stable network structure which results in viscosity increase; therefore, it is known as a viscoelastic surfactant (VES). Viscoelastic characteristics of VES are attributed to worm-like micelles that entangle together to create transient network structures. A growing number of studies examine highly elongate cylinders (worm-like) VES application in fracturing [6]. The fluid’s viscosity is affected by the pH and surfactant concentration [16]. 

**Figure 1:**
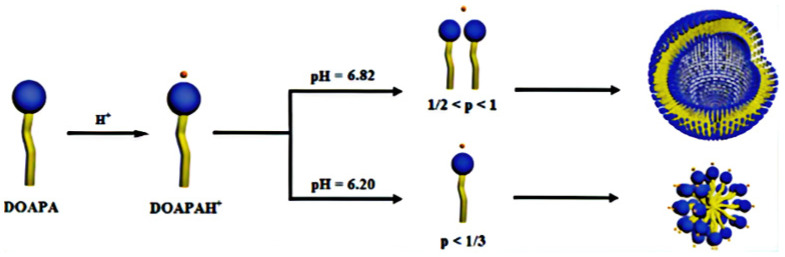
pH reduction changes vesicle to a spherical micelle [19].

**Figure 2:**
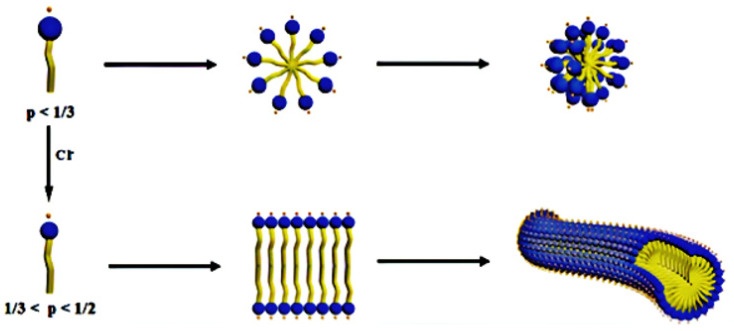
Cl^−^ increase transfer spherical micelle to a worm-like micelle [19].

The pH affects the viscosity of VES fluid in a narrow window; usually, decreasing the pH level results in a higher viscosity. Moreover, adding CO_2_ helps in the transition of the spherical VES to worm-like micelles because it lowers the solution pH by the formation of carbonic acid. With the addition of hydrochloric acid (HCl), vesicles gradually transform into micelles and then into worm-like micelles. The vesicles are also sensitive to the pH and Cl^−^ concentration which may result in worm-like micelles. Typically, the packing parameter *p* is used to describe the self-assembly behavior of micelles. This is a geometric quantity (*GQ*) denoted by:

(1) $$GQ=\frac{V}{al}$$

where *a* is the effective headgroup area, *V* denotes the volume of the most lipophilic chain, and *l* is the effective length. When *p* < 1/3 is reached, spherical micelles develop; at 1/3 < *p* < 1/2, the worm-like micelles are formed, and at 1/2 < *p* < 1, the vesicles or bilayers micelles are formed. It is possible to obtain vesicles or bilayers. The structure depicted in Figure 1 is the smallest unit in these structures, with the *N*,*N*-dimethylolamidopropylamine (DOAPA) [18] being the smallest unit, while the DOAOAH^+^ is when hydrogen is added. Additionally, the Figure demonstrates how a little variation in pH results in the formation of a distinct structure and changes the vesicles to spherical micelles. Additionally, when the Cl^−^ ions are added, the spherical micelles can transform into worm-like micelles; Figure 2 illustrates this transmission [19].

## 2. VES Applications

### 2.1. VES as Acid Diverter

In tight carbonate formations, acid could be injected to enhance productivity. The acid creates wormholes (i.e., high permeability structures) to bypass damage in carbonate formation. It flows to the high permeable zones leaving the tight sections under-stimulated. Therefore, acid diversion is implemented to ensure all intervals are stimulated. The method is based on sealing off the high permeable zones temporarily and transporting acid elsewhere. Poor diversion leads to partial damage removal and results in uneconomical acid pumping volumes. It’s common to use mechanical (like plugs) and chemical (like foams, polymer gels, and salts) diversion methods [16,20]. VES-based fluid was introduced as a diverting system. When the acid reacts with the carbonate, the pH increases, and the VES gels in-situ, forcing acid to self-divert to the low permeability zones [20,21]. 

VES as diverting materials was experimented with a 20-inch core for the first time in the research [22]. It was observed that the wormhole shape was more torturous than it was during normal acidizing. The use of electrolytes with VES as a diverting system reduced the need for high surfactant concentration [16]. Al-Sadat et al. (2014) studied the rheological behavior of zwitterionic anionic surfactant used in acid stimulation [14]. A VES concentration of 7.5 wt.% showed the highest elastic strength in freshwater. A CaCl_2_ concentration of 22 wt.% in brine provided the highest elasticity. MgCl_2_ and CaCl_2_ showed an increase in solution elasticity more than NaCl, KCl, and NH_4_Cl [14]. For VES micellar in CaBr_2_ and CaCl_2_ brines, adding MgO or ZnO nanoparticles increased thermal stability and viscosity at varied shear speeds [23].

### 2.2. VES in Enhanced Oil Recovery (EOR)

A substantial amount of oil remains untapped during waterflooding as water prefers the path with the least resistance [23]. Sweep efficiency during waterflooding could be improved using polymers, which increases fluid viscosity and elasticity. Similarly, VES could be utilized, which possesses extraordinary viscoelastic characteristics due to the formation of complex worm-like micelles. This property raises the viscosity of the displacing fluid while simultaneously decreasing the IFT between oil and water [24]. Janjua et al. (2020) conducted core flooding experiments to recover heavy oil from carbonate rock using VES mixed with diethylene triamine pentaacetic acid (DTPA) chelating agent. They studied viscosity and interfacial tension at different concentrations, temperatures, and times. It resulted in an efficient displacement and reduced the interfacial tension [23]. Short-term testing of the VES samples at high temperatures showed strong thermal stability; however, aging the samples at high temperatures increased their IFT over time [23]. Morvan et al. evaluated a VES-based fluid for chemical enhanced oil recovery (CEOR). The viscosity of the formulated fluid is insignificantly affected by the presence of brine concentration when tested in low salinity and synthetic seawater [25].

Surfactant-stabilized N_2_/CO_2_ water-based foams are the most extensively employed in foamed EOR. Sun et al. (2019b) studied potential VES formulations and their performance under harsh reservoir conditions. The low interfacial tension of the foaming surfactants performs well in fractured/tight reservoirs, while CO_2_-switchable surfactants work in high temperatures carbonate reservoirs. If the surfactant contains both the cationic and anionic groups, it performs well as both vesicles and worm-like micelles to enhance the foam stability [26].

### 2.3. VES in Hydraulic Fracturing

Fracturing fluids require high viscosity, usually achieved utilizing polymers such as cellulose, xanthan gum, and polyacrylamide. These polymers can leave heavy residues within the proppant pack, resulting in reduced fracture permeability. Moreover, many polymers cannot perform well in high-temperature environments due to their fast degradation. VES can replace polymer in hydraulic fracturing because of its high viscosity during injection and the low viscosity during flow back. Worm-like micelles must be produced to enhance a VES-based fracturing fluid viscosity. Certain types of surfactants may form spherical micelles instead of worm-like micelles as a result of their packing parameter. Hence, many surfactants do not have the potential to be transformed into VES surfactants [17]. VES can carry proppant effectively at a lower viscosity and can deliver proppant deep into the formation. Besides, it flows back without gel breakers, and it leaves minimum residue within the formation. Despite this, the VES has several disadvantages such as low stability at high shear rates and temperatures, low tolerance for salts, high cost, and requiring more significant concentrations than polymer-based fluids. Additionally, the single head-tail VES requires a high critical micelle concentration, which results in greater molecular weight than the polymer. The Gemini surfactant was developed to address this, which combines two single head-single tail surfactants connected by a spacer group. The spacer group prevents electrostatic repulsion between hydrophilic groups [6,27]. 

Tri-cationic surfactant contains three single-chains, and a spacer group acts as a thickener. Apart from breaking after two hours, this VES demonstrated excellent viscoelastic characteristics and proppant suspending capabilities. Additionally, when the VES content was between 3–5 wt.%, it demonstrated good thermal stability between 140 and 180 °C [27]. Schlumberger pioneered the use of VES in the formulation of fracturing fluid in 1997 [6,27]. They compared VES and polymer-based treatments and discovered that VES resulted in much greater initial production. However, since polymers are less costly than VES, they are used more often in fracturing operations. 

### 2.4. VES Mixed with Polymers

Ideally, gelled fracturing fluid should break at the end of the treatment; instead, residual polymer precipitates and damage the formation. Although VES fracture fluid is suitable at high salinity and high-temperature environments, higher viscosity values with lower VES concentrations are required in these conditions. The addition of polymers to the VES can enhance the stability and viscosity of the fluid. The combined VES-polymer fluid system performs better than either VES or polymer alone [6]. In this mix, the surfactants are used to improve the dispersion, wetting, and suspension properties, while the polymers are used to enhance the rheological properties. This combination can result in electrostatic interaction when the polymer and surfactant are oppositely charged and hydrophobic interaction between the hydrophobic parts of the polymer and the surfactant. The main interaction is the hydrophobic one, and the complementary charged polymers enhance the aggregate’s stability. The addition of polymer can change the surface tension and the critical micelle concentration value. If the polymer has an opposite charge to the surfactant, it reduces the critical micelle concentration value of the surfactant [17]. Beheshti et al. (2006) studied the mixtures of anionic and neutral HEC polymer with anionic and cationic surfactants (SDS and CTAB, respectively). Weak interaction between the anionic HEC and anionic SDS is noted, while anionic HEC with cationic CTAB reaction formed a large polymer–surfactant association, which positively affected the rheology [28]. In another study, the oppositely charged surfactant was added to cationic HEC polymers. They showed phase separation at certain surfactant concentrations. When compared to a pure cationic HEC, a stronger viscoelastic property of the mixture resulted [29].

### 2.5. Cellulose Functional Groups

Because cellulose has high crystallinity, it is insoluble in water; nevertheless, treating it with a hydrophilic functional group transformed it to water-soluble. In 1912, nonionic methylcellulose (MC) was synthesized by reacting dimethyl sulfate with cellulose in a basic solution; six years later, water-soluble ionic CMC was synthesized. It is vital to understand the structure of cellulose to change it. Later, hydroxyethyl cellulose (HEC), hydroxypropyl cellulose (HPC), hydroxypropyl methylcellulose (HPMC), and sulfo-ethyl cellulose were synthesized. The acrylamide, acrylonitrile, and acrylic acid are hydrophilic vinyl monomers grafted onto CMC or HEC. The variety of characteristics of these polymers is determined by the number of functional groups and their placement in the polymer, as well as the molecular weight of the polymer. Each polymer derivative has a distinct use. Recently, the polymerization industry has seen the introduction of well-defined polymers with regulated structure and molecular weight [30,31]. Typical processes for the synthesis of non-ionic cellulosic may be employed to add ionic groups while maintaining the polymer chain intact. Esterification, oxidation, grafting, etherification, and nucleophilic substitution are all examples of these processes. While etherification may be used to synthesize anionic and cationic polysaccharides (e.g., CMC), this reaction can be used for HEC with sodium chloroacetate to form sodium carboxymethyl hydroxyethyl ether of cellulose (Na-CMHEC) [32]. More cellulose-based functional materials are being developed and used for gels which are used in different applications, i.e., hydroxypropyl methyl celluloses (HPC) [33]. 

In this paper, we investigate the influence of different polymer additives on the viscosity of the VES fluid depending on their charge and functional groups while maintaining the high-temperature stability and good compatibility of the fluid at high salinity. The polymer can interact positively with the surfactant molecule to improve rheology. Therefore, it can increase the viscosity of the VES fluid while allowing for a reduction in the VES load in the fluid, which results in cost reduction. Significant increases in viscosity were observed when a small amount of the polymer was added. The viscosity increased because of solution interaction and did not involve crosslinking of polymers. Also, different tests were performed to compare the efficiency of two types of polymers; anionic|(CMC) and neutral (HEC) with VES. 

## 3. Materials and Methods

### 3.1. Materials 

**Figure 3:**
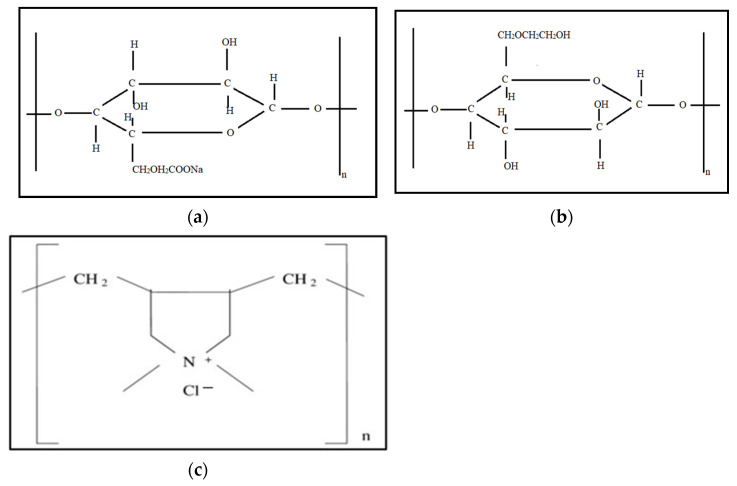
Structures of (**a**) Carboxymethyl cellulose (CMC), (**b**) Hydroxyethyl cellulose (HEC), and (**c**) Diallyl dimethylammonium chloride (DADMAC) [34,35].

Different polymers were tested with zwitterionic VES, which are carboxymethyl cellulose (CMC) polymer, hydroxyethyl cellulose (HEC), and a poly diallyl dimethylammonium chloride (DADMAC) polymer. Figure 3 shows the structure of CMC and HEC. Polymer selection was based on their compatibility and type where CMC is anionic, and HEC is neutral; they are both based on cellulose while DADMAC is a cationic polymer. These polymers were mixed at different concentrations with the VES to form variants of the fracturing fluid with different rheology and stability. Calcium chloride (CaCl_2_) salt was used to prepare the VES solutions. The distilled water was applied in all freshwater experiments. These variations were also tested with seawater.

### 3.2. Experimental Design

Chandler rheometer 5550 HPHT was used for the rheology tests of different novel combinations of polymers and VES at a 100 1/s shear rate. First, we prepared the calcium chloride solution, then the CMC, HEC, or DADMAC (which is named DP in this paper) were added. The specific polymer was mixed for 5 min with CaCl_2_ then the VES was added. The performed tests in this research consisted of shearing the fluid while ramping the temperature to 171.1 °C. Also, the prolonged viscosity tests at fixed temperatures (i.e., 93.3 °C, 143.3 °C) were performed. The viscosity tested was plotted at different concentrations of VES, CaCl_2_, and polymers. Table 1 shows the design and the list of the experiments, as it illustrates the different chemical variations at different conditions. The CMC and HEC polymers are prepared and tested with freshwater, while the newly designed DP polymer results are with the SW.

## 4. Results and Discussion

### 4.1. Influence of CMC on VES Fluid

In this set of experiments, different concentrations of CMC were tested (i.e., 0.1%, 0.5%, and 1%) with 9% (*v*/*v*) VES. The experiments were conducted within two hours, and the temperature was increased gradually from 23.9 to 176.7 °C at 1.4 °C per min ramp rate. All solutions were prepared in 30% (*w*/*v*) CaCl_2_, and the rheology was tested at a constant 100 1/s shear rate.

**Figure 4:**
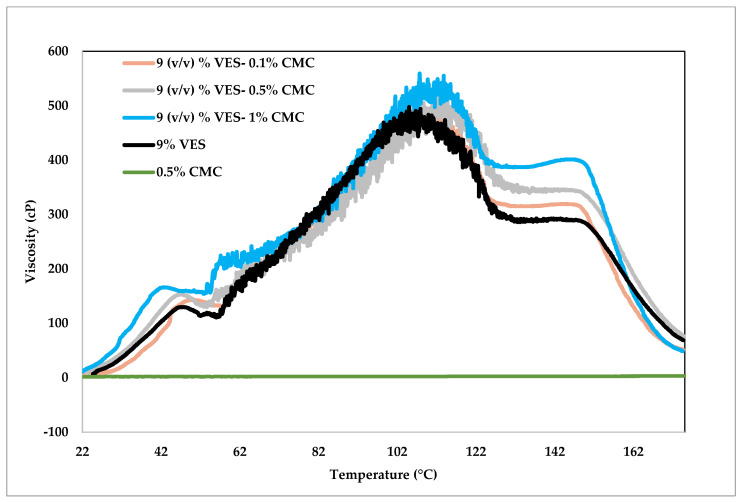
Viscosity change with respect to temperature at 100 1/s shear rate, 9% (*v*/*v*) VES and different CMC polymer concentrations.

**Figure 5:**
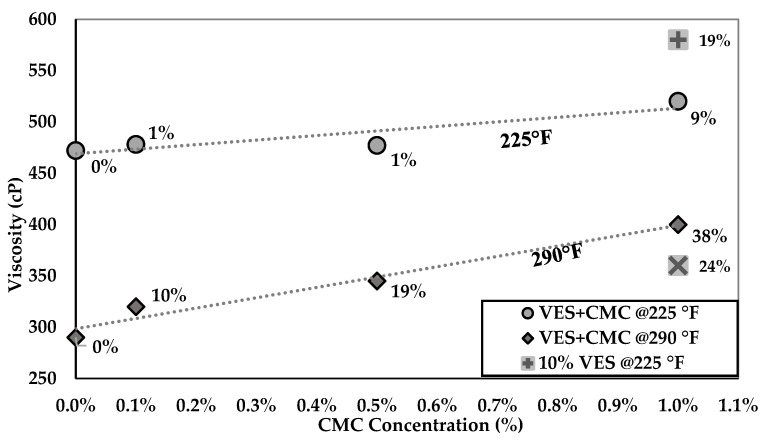
The increase of VES viscosity with different CMC concentration at the selected temperatures.

Figure 4 shows the impact of temperature on the rheology behavior of VES-based fluids. The purple line shows the rheology test of 0.5% CMC in CaCl_2_ solution without any additives, indicating low viscosity values of 2–3 cP at all temperatures. On the other hand, the VES fluid viscosity increased with temperature up to 110 °C, then dropped steadily by nearly 40% and stayed constant between 126.7 °C and 148.9 °C, and sharply declined after that. The application of VES in operations should be carefully revised if the temperature is higher than 148.8 °C due to the surfactant stability limitations. The addition of CMC to VES did not significantly impact the rheology profile with respect to temperature but resulted in variation of viscosity magnitudes. The impact is more pronounced at higher temperatures, as the figure indicates. Table 2 shows, for comparison, the extracted viscosity data of all fluid types at 107.2 °C and 143.3 °C. The base case in the table was 9% (*v*/*v*) VES without any addition of CMC. One might notice that the increase in CMC concentration resulted in increased viscosity at both temperatures. The increase in viscosity was more pronounced at 143.3 °C, where the 1% CMC increased the viscosity by 38%. The table shows that increasing the concentration of VES by 1% (*v*/*v*) increased the viscosity more than that achieved by adding 1% CMC (see the last two rows) at 107.2 °C; nevertheless, the behavior is reversed at 143.3 °C. This fluid behavior could be used to reduce the concentration of VES in favor of CMC at high temperatures, resulting in cost reduction. Figure 5 visually shows the comparison provided in Table 2.

**Figure 6:**
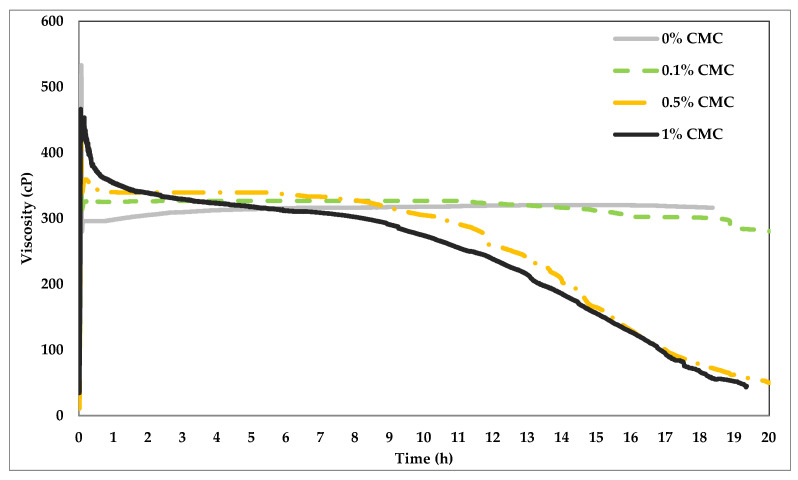
The viscosity changes with respect to time for 9% (*v*/*v*) VES at different CMC concentrations at 300 F.

During hydraulic fracturing, a higher viscosity is preferred during injection (2–3 h and a reduced viscosity during flow back. Figure 6 shows the rheology test of the same fluid systems at constant temperature (148.9 °C) and shear rate (100 1/s). The figure indicates that the VES alone is very stable with low viscosity fluctuations during 20 h of constant shearing. The addition of the CMC, nevertheless, impacted both the stability and viscosity of the fluid system. At early shearing times, the higher the concentration of CMC, the higher the viscosity values. However, the rate of viscosity decline is also positively related to the CMC concentration. It could be noticed that the CMC, especially at high concentration, resulted in breaking the viscosity of the fluid. CMC being thermally unstable at this temperature, degraded with time and which potentially impacted the wormlike micellar structure of the VES, leading to a progressive decrease in viscosity of the fluid. The fluid after 20 h of heating was visually observed to change to dark brown color from its initial white color when freshly prepared at room temperature. The behavior of VES with 1% CMC is favorable for hydraulic fracturing application where higher viscosity is required to create fracture and place the proppant and then drops down for efficient flowback. However, we would like to emphasize that this fluid requires an additional breaker additive for a more efficient flowback. Specifically, to obtain much lower viscosity and with better control over the time of breaking while maintaining the viscosity gain during the initial hour.

### 4.2. Influence of Hydroxyethyl Cellulose (HEC) on VES Fluid

**Figure 7:**
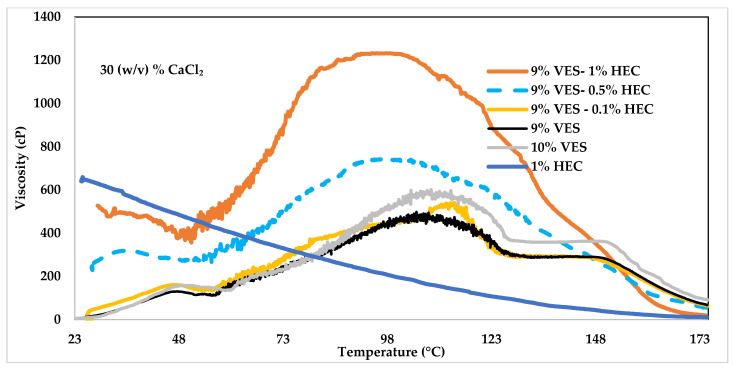
Viscosity change with respect to temperature at 100 1/s shear rate, 9% (*v*/*v*) VES fluid prepared with 30% (*w*/*v*) CaCl_2_ in fresh water and 1% HEC polymer concentrations.

The following experiments evaluated various concentrations of HEC (i.e., 0.5%, and 1%) mixed with 9% (*v*/*v*) VES. As baseline experiments, 9% (*v*/*v*) VES, 10% (*v*/*v*) VES, and 1% HEC were tested individually. The experiments lasted less than two hours while the temperature increased from 23.9 to 176.7 °F at 1.4 °C per min ramp rate. All solutions were prepared in 30% (*w*/*v*) CaCl_2_, and their rheology was determined at a constant shear rate of 100 1/s. The effect of temperature on the rheology of VES-based fluids when using HEC is illustrated in Figure 7. The green line indicates that at temperatures less than 143.3 °C, the 9% (*v*/*v*) VES + 0.5% HEC has higher viscosity than 9% or 10% (*v*/*v*) VES. However, with the addition of HEC, the onset of the rapid decrease in viscosity begins to occur earlier than that observed with pure VES fluids. In fact, the constant viscosity state was observed between 126.7–148.9 °C for the baseline VES fluids cease to occur in the presence of HEC. Consequently, at 143.3 °C, the viscosity values with HEC began to drop less than that was observed with the baseline 9% VES fluids. The pure 1% HEC started with a viscosity of 650 cP at room temperature but rapidly decreased to less than 50 cP at 148.9 °C. The dark green line represents 9% (*v*/*v*) VES + 1% HEC; the viscosity exceeded 1200 cP between 93.3 and 107.2 °C. Although the viscosity decreased steadily after 107.2 °C, it maintained high values compared to 9% and 10% (*v*/*v*) VES until 137.8 °C. One might conclude that adding higher HEC concentrations to VES leads to significantly higher viscosity. It is worth noting that, unlike CMC, HEC have a viscosifying effect. However, the increase in viscosity did not appear to be an additive effect of but rather a synergetic effect of the formulation. 

### 4.3. Influence of CaCl_2_ on VES 

**Figure 8:**
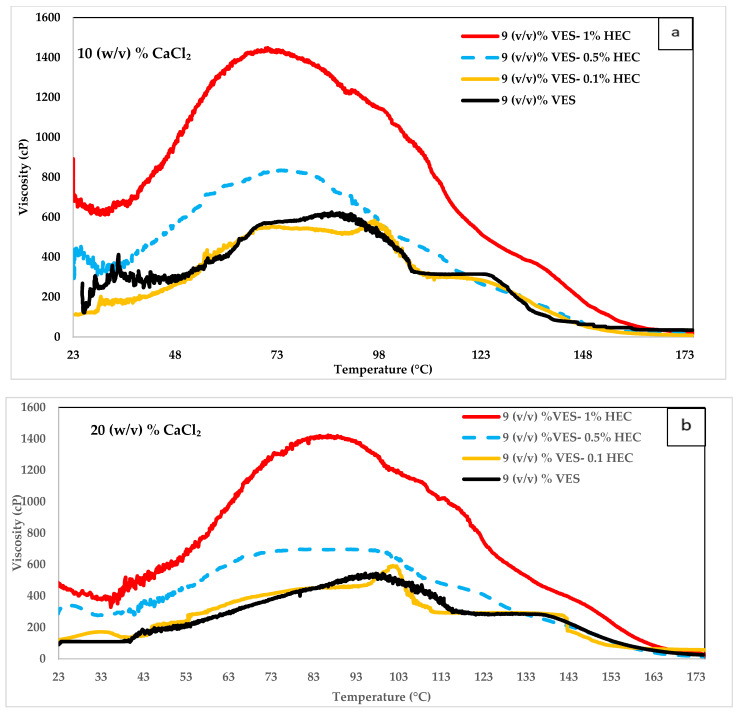
VES + HEC viscosity variation with respect to temperature at 10% (**a**) and 20% (**b**) CaCl_2_ concentrations.

CaCl_2_ concentrations of 10%, 20%, and 30% (*w*/*v*) in 9% (*v*/*v*) VES was sheared at increasing temperatures, with and without HEC concentrations of 0.1%, 0.5%, and 1%. The resulting rheology demonstrates the effect of CaCl_2_ concentration on viscosity. Figure 8a,b shows the rheology behavior of VES when added to 10% (*w*/*v*) CaCl_2_ and 20% (*w*/*v*) CaCl_2_, respectively. We compared these figures to Figure 7, which shows the rheology of VES when it was added to 30% (*w*/*v*) CaCl_2_. At temperatures above 93.3 °C, increasing CaCl_2_ concentration results in increased viscosity values and high-temperature stability (shift of peak viscosity). However, at temperatures less than 93.3 °C, the viscosity is higher at lower CaCl_2_ concentrations. Hence, the concentration of CaCl_2_ can be optimized based on the temperature encountered during operations.

In all three figures, when comparing the VES at different HEC concentrations, we found that the higher the HEC concentration, the higher the viscosity except at temperatures above 160 °C. At very high temperatures above 160 °C, all HEC concentrations with 9 (*v*/*v*) % VES in the three CaCl_2_ concentrations have similar viscosity values. In all CaCl_2_ concentrations, adding 0.1% HEC to 9 (*v*/*v*) % VES did not improve the viscosity significantly. The mixture containing 30 (*w*/*v*) % CaCl_2_, 9 (*v*/*v*) % VES, and 0.1% HEC have better viscosity than the 10 (*v*/*v*) % VES at temperatures lower than 93.3 °C. 

### 4.4. Temperature Impact on VES Systems

**Figure 9:**
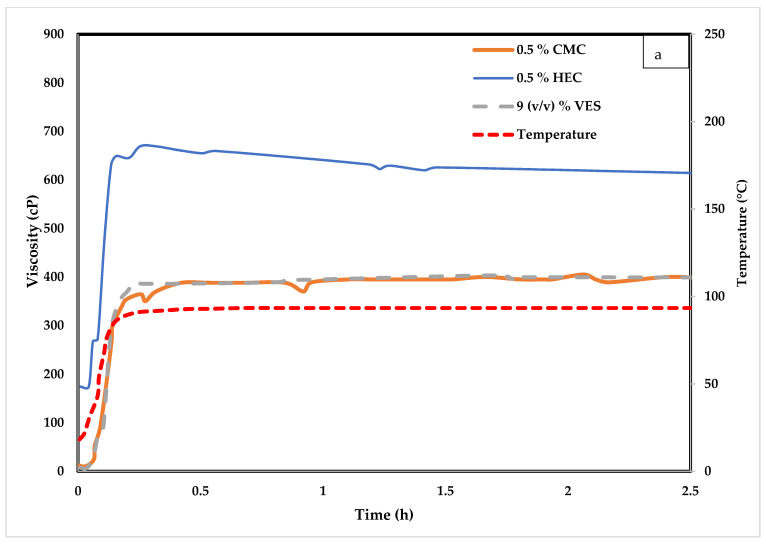

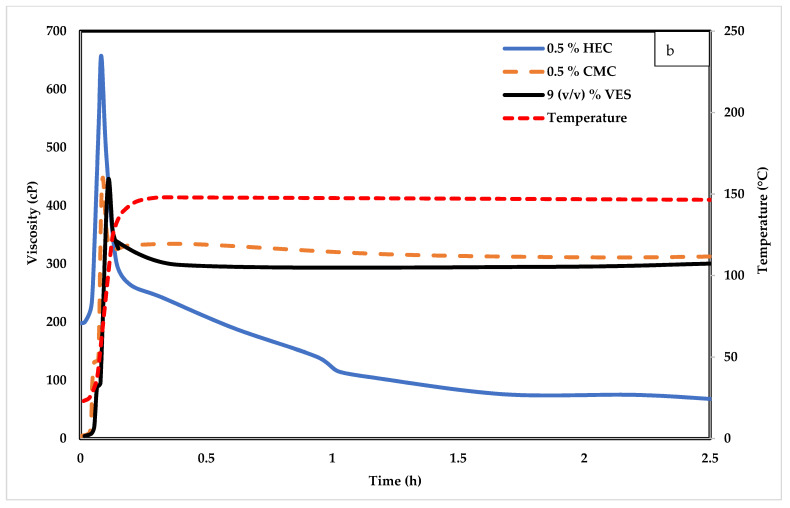
CMC and HEC effect on VES at (**a**) 200 °F and (**b**) 300 °F.

Figure 9a, shows the rheology test for 9% (*v*/*v*) VES, 9% (*v*/*v*) VES with 0.5 HEC, and 9% (*v*/*v*) VES with 0.5% CMC at 30% (*w*/*v*) CaCl_2_ at constant temperature (93.3 °C). Figure 9b shows the tests for the same fluid systems at constant temperature (148.9 °C). The tests in both temperatures were performed for 2 h and 30 min at a shear rate of 100 1/s. At 93.3 °C, the viscosity starts low and stays stable at slightly less than 400 cP for the 9% (*v*/*v*) VES and 9% (*v*/*v*) VES with 0.5% CMC. The viscosity is also stable for the 9% (*v*/*v*) VES with 0.5% HEC, but the viscosity reached 650 cP. At 148.9 °C (see Figure 9b), a significant viscosity drop is observed for the 9% (*v*/*v*) VES with 0.5% HEC; in less than 10 min, it dropped from 650 cP to less than 300 cP and reached 100 cP in 90 min. On the other hand, the 9% (*v*/*v*) VES and 9% (*v*/*v*) VES with 0.5% CMC dropped from 450 cP to 300 cP in the first 10 min and remained stable at this value for the remaining test time. This illustrated that HEC destabilizes the system at 148.9 °C compared to CMC but has a better synergetic effect with VES at lower temperatures. The difference can be attributed to the thermal stability of HEC and CMC at high temperatures and to the difference in the interaction between the non-ionic HEC and anionic CMC, respectively, with the micellar structures of the zwitterionic VES. Further investigation is necessary to determine the influence of each of these factor on the observed viscosity profiles. 

### 4.5. Influence of Poly Diallyldimethylammonium Chloride (DADMAC) on VES Fluid

**Figure 10:**
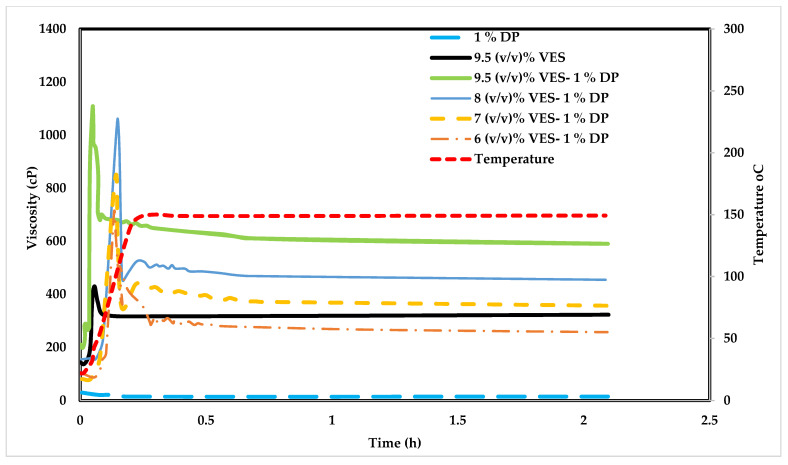
Different concentrations of VES Fluid prepared with 30% *w*/*v* CaCl_2_ in sea water, at 1% of DP polymer.

Figure 10 shows the viscosity change with time at different temperatures for the 9.5% (*v*/*v*) VES, 1% DADMAC (DP), 9.5% (*v*/*v*) VES with 1% DP, 8% (*v*/*v*) VES with 1% DP, 7% (*v*/*v*) VES with 1% DP, and 6% (*v*/*v*) VES with 1% DP, at 30% (*w*/*v*) CaCl_2_. Different from previous experiments, this polymer was tested in seawater. This polymer showed better thermal stability and did not degrade with time at 148.9 °C. The following experiments were to test DADMAC polymer when added to VES, which was prepared with 30% (*w*/*v*) CaCl_2_ in seawater. Figure 10 illustrates that the system did not break at high temperatures, as was observed with 0.5% HEC and 1% CMC. The viscosity doubled by adding 1% DADMAC to the VES (compare the blue to the maroon lines). It is of note that the DP alone did not generate high viscosity when prepared with CaCl_2_. However, a mix of 1% of this polymer with 6% or 7% (*v*/*v*) VES can result in similar rheology as with the 9.5% (*v*/*v*) VES. 

**Figure 11:**
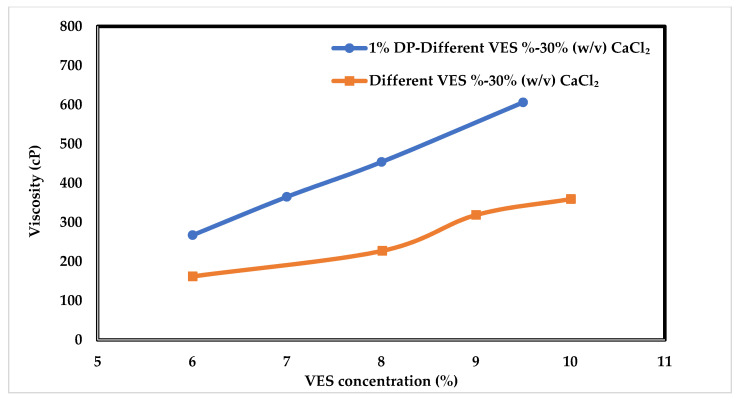
Viscosity variation with VES concentration at 300 °F.

Figure 11 shows the viscosity of VES with and without adding 1% DP polymer at 148.9 °C. The rate of viscosity increase with respect to VES concentration is higher when adding the DP polymer. It is noted that the 6% (*v*/*v*) VES with 1% DP can give almost a similar result as the 9% (*v*/*v*) VES without the polymer. This comparison was performed to reduce the volume requirement of the expensive VES in favor of less expensive polymers. Comparing these results with those obtained from cellulose derivatives, it can be noticed that the viscosity increase with DADMAC is similar to that observed with the nonionic HEC polymer. While the viscosity enhancement with HEC was mainly at temperatures below 132.2 °C, DADMAC, provided high enhancement even at 148.9 °C. Unlike HEC, only DADMAC solution has very low viscosity, in the range of 12–15 cP. Hence the viscosity enhancement was purely due to the synergistic interaction of the VES micelles with the cationic polymer. Being thermally more stable than the cellulose derivatives at high temperatures, DADMAC continued to provide enhancements at high temperatures.

#### The Impact of CaCl_2_ on VES

**Figure 12:**
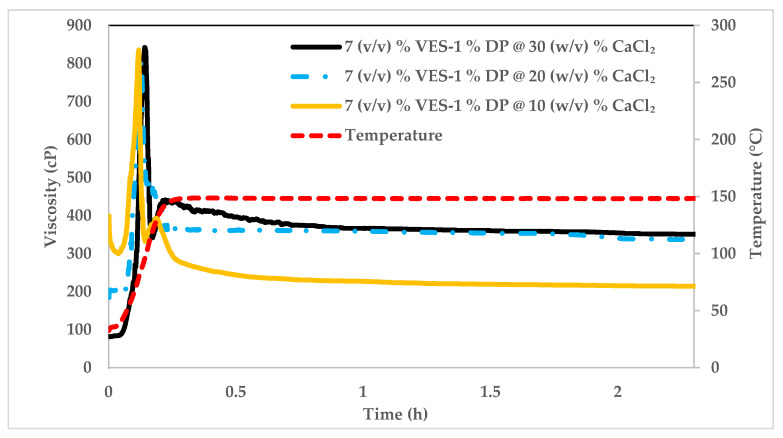
Viscosity profile of fluid with 7% (*v*/*v*) VES, 1% DP polymer only and 10%–30% (*w*/*v*) CaCl_2_ in sea water solution. Measurement done at constant shear rate of 100 1/s.

Figure 12 shows the rheology of 7% (*v*/*v*) VES with DP polymer sheared at 100 1/s and 148.9 °C. The mixture was prepared with three CaCl_2_ concentrations of 10%, 20%, and 30%, respectively. The resulting rheology demonstrates the effect of CaCl_2_ concentration on rheology. We can notice that at 148.9 °C, increasing CaCl_2_ concentration increases the viscosity at later times but lowers it at initial shearing times (first five minutes). Increasing the concentration of CaCl_2_ from 10% to 20% significantly impacts the viscosity values. Nevertheless, an increase from 20% to 30% does show any advantage in terms of viscosity increase at later shearing times.

## 5. Conclusions

VES was introduced to the industry due to its favorable viscoelastic properties and less damaging impact; nevertheless, it is costly and only effective at high concentrations compared to polymers. This research targeted optimizing VES concentration by introducing different polymers such as CMC, HEC, and DADMAC at different temperatures and CaCl_2_ concentrations. The main outcomes of this study are:

1. The study shows that polymers can be used to reduce the VES concentration and improve the rheology.
2. Different concentrations of polymers were added to VES and compared to pure VES rheology.
3. CaCl_2_ concentration can regulate the VES viscosity at different temperatures. For instance, at low temperatures, the low CaCl_2_ concentrations can yield larger viscosities and vice versa for high temperatures.
4. At different CaCl_2_ concentrations, the VES system viscosity became stable after the first 30 min.
5. The system containing 9 (*v*/*v*) % VES and 0.1% HEC has a superior viscosity than the one containing 10 (*v*/*v*) % VES at temperatures lower than 93.3 °C in 30 (*w*/*v*) % CaCl_2_ solution.
6. The systems containing 9 (*v*/*v*) % VES, 9 (*v*/*v*) % VES + 0.1% HEC, and 9 (*v*/*v*) % VES + 0.5% HEC have very similar viscosity values above 160 °C.
7. A synergistic effect of CMC, HEC, and DADMAC on the viscosity of VES was observed. While HEC in freshwater provides a higher viscosity at lower temperatures; CMC has relatively better thermal stability. The DADMAC polymer showed excellent viscosity and stability in seawater.
8. Charge of the polymer apparently has a significant impact in influencing the rheology of the VES fluid.

## Figures and Tables

**Table 1:** The viscosity experiments performed at high temperatures.

Test Type	Tested Polymer/Chemical	VES Conc. (*v*/*v*) %	Polymer Conc. (*v*/*v*) %	CaCl_2_ Conc. (*w*/*v*) %	Temperature °C
Viscosity vs. Temperature	VES	9	0	10	26.7–171.1
		9	0	20	26.7–171.1
		9	0	30	26.7–171.1
		10	0	30	26.7–171.1
	CMC	0	0.5	30	26.7–171.1
		9	0.1	30	26.7–171.1
		9	0.5	30	26.7–171.1
		9	1	30	26.7–171.1
	HEC	9	0	10	26.7–171.1
		9	0.1	10	26.7–171.1
		9	0.5	10	26.7–171.1
		9	1	10	26.7–171.1
		9	0	20	26.7–171.1
		9	0.1	20	26.7–171.1
		9	0.5	20	26.7–171.1
		9	1	20	26.7–171.1
		9	0	30	26.7–171.1
		9	0.1	30	26.7–171.1
		9	0.5	30	26.7–171.1
		9	1	30	26.7–171.1
		0	1	30	80–340
Viscosity vs. Time (Fixed Temperature)	DP	0	1	30	93.3
		6	1	30	93.3
		7	1	30	93.3
		0	1	30	148.9
		8	1	30	148.9
		7	1	30	148.9
		7	1	20	148.9
		7	1	10	148.9
		6	1	30	148.9
		9	0	30	148.9
	CMC	9	0.5	30	93.3
		9	0.1	30	148.9
		9	0.5	30	148.9
		9	1	30	148.9
	HEC	9	0.5	30	93.3
Viscosity vs. polymer concentration	VES	10	0	30	148.9
		9	0	30	148.9
		8	0	30	148.9
		6	0	30	148.9
	CMC	9	0.1	30	107.2 & 143.3
		9	0.5	30	107.2 & 143.3
		9	1	30	107.2 & 143.3
	DP	9.5	1	30	148.9
		8	1	30	148.9
		7	1	30	148.9
		6	1	30	148.9

**Table 2:** Viscosity variation for different fluids at 107.2 °C and 143.3 °C.

Fluid Type	Viscosity at 107.2 °C	Viscosity at 143.3 °C	% Increase at 107.2 °C	% Increase at 143.3 °C
0.5% CMC	0	2.6	--	--
9% (*v*/*v*) VES	472	290	--	--
9% (*v*/*v*) VES + 0.1% CMC	478	320	1%	10%
9% (*v*/*v*) VES + 0.5% CMC	477	345	1%	19%
9% (*v*/*v*) VES + 1% CMC	520	400	9%	38%
10% (*v*/*v*) VES	580	360	19% (+)	24% (X)

## Abbreviations

VES	viscoelastic surfactants
CMC	carboxymethyl cellulose
HEC	hydroxyethyl cellulose
DADMAC	poly diallyl dimethylammonium chloride
HPHT	high pressure high temperature
PFOS	perfluoro octane sulfonate
DOSS	dioctyl sodium sulfosuccinate
Triton X-100	ethylene glycol octyl phenol ethers
FMES	fatty methyl ester sulfonate
CTAB	cetyltrimethylammonium bromide
CTAC	cetyltrimethylammonium chloride
SDS	Sodium Dodecyl Sulfate
CHAPS	3-[(3-Cholamidopropyl) dimethyl ammonio]-1-propane sulfonate
TEM	transmission electrical microscopy
SEM	scanning electron microscope
AFM	atomic force microscope
DOAPA	*N*,*N*-dimethylolamidopropylamine
DTPA	diethylene triamine pentaacetic acid
